# A unique androgen excess signature in idiopathic intracranial hypertension is linked to cerebrospinal fluid dynamics

**DOI:** 10.1172/jci.insight.125348

**Published:** 2019-03-21

**Authors:** Michael W. O’Reilly, Connar S.J. Westgate, Catherine Hornby, Hannah Botfield, Angela E. Taylor, Keira Markey, James L. Mitchell, William J. Scotton, Susan P. Mollan, Andreas Yiangou, Carl Jenkinson, Lorna C. Gilligan, Mark Sherlock, James Gibney, Jeremy W. Tomlinson, Gareth G. Lavery, David J. Hodson, Wiebke Arlt, Alexandra J. Sinclair

**Affiliations:** 1 Institute of Metabolism and Systems Research, University of Birmingham, Edgbaston, Birmingham, United Kingdom.; 2 Centre for Endocrinology, Diabetes and Metabolism, Birmingham Health Partners, Edgbaston, Birmingham, United Kingdom.; 3 Department of Neurology, and; 4 Birmingham Neuro-Ophthalmology Unit, Ophthalmology Department, University Hospitals Birmingham NHS Foundation Trust, Birmingham, United Kingdom.; 5 Department of Endocrinology and Diabetes Mellitus, Tallaght Hospital, Tallaght, Dublin, Ireland.; 6 Oxford Centre for Diabetes, Endocrinology and Metabolism, National Institute of Health Research (NIHR) Biomedical Research Centre, University of Oxford, Churchill Hospital, Oxford, United Kingdom.; 7 Centre of Membrane Proteins and Receptors, University of Birmingham, and University of Warwick, Birmingham, United Kingdom.

**Keywords:** Endocrinology, Neuroscience, Neurological disorders

## Abstract

Idiopathic intracranial hypertension (IIH) is a condition of unknown etiology, characterized by elevated intracranial pressure frequently manifesting with chronic headaches and visual loss. Similar to polycystic ovary syndrome (PCOS), IIH predominantly affects obese women of reproductive age. In this study, we comprehensively examined the systemic and cerebrospinal fluid (CSF) androgen metabolome in women with IIH in comparison with sex-, BMI-, and age-matched control groups with either simple obesity or PCOS (i.e., obesity and androgen excess). Women with IIH showed a pattern of androgen excess distinct to that observed in PCOS and simple obesity, with increased serum testosterone and increased CSF testosterone and androstenedione. Human choroid plexus expressed the androgen receptor, alongside the androgen-activating enzyme aldoketoreductase type 1C3. We show that in a rat choroid plexus cell line, testosterone significantly enhanced the activity of Na^+^/K^+^-ATPase, a surrogate of CSF secretion. We demonstrate that IIH patients have a unique signature of androgen excess and provide evidence that androgens can modulate CSF secretion via the choroid plexus. These findings implicate androgen excess as a potential causal driver and therapeutic target in IIH.

## Abstract 2

Women with idiopathic intracranial hypertension have a unique androgen excess signature that is associated with modulation of cerebrospinal fluid secretion dynamics.

## Introduction

Idiopathic intracranial hypertension (IIH) is a chronic and disabling condition characterized by elevated intracranial pressure (ICP) (1). Patients have reduced quality of life due to chronic headaches and papilledema-induced visual loss (permanent in 25% of patients) (2, 3). Predominantly occurring in areas of socioeconomic deprivation, IIH incidence is rising rapidly in line with the global epidemic of obesity (2 per 100,000 in 2002 to 5 per 100,000 in 2016), driving escalating health care costs (predicted at ≤450 million dollars per year by 2030) (4). IIH is overwhelmingly a disease of obese women of reproductive age (5, 6), and endocrine disturbances have been hypothesized to play a pathogenic role (7, 8). However, IIH etiology remains unexplained (3); identifying the cause of IIH was determined as the most important research question by IIH patients in a recent research priority–setting exercise (3).

IIH shares phenotypic characteristics with polycystic ovary syndrome (PCOS), in which androgen excess is a defining feature (9). Studies have suggested that the prevalence of PCOS is increased in IIH, as compared with the background population (10, 11), and case reports have described the onset of IIH in patients receiving androgen treatment when undergoing female-to-male sex reassignment (12). Hyperandrogenism has been identified in younger onset cases of IIH (13). However, the androgen phenotype of women with IIH has not been studied in detail or compared with the androgen profile in PCOS. Androgen receptors are noted in the choroid plexus (14, 15), but there are no mechanistic studies examining their potential impact on cerebrospinal fluid (CSF) secretion.

We aimed to delineate the androgen phenotype of women with active IIH and to explore the potential role of androgens in disease pathogenesis. To this end, we have performed complementary in vivo and *ex vivo* investigations, comprehensively examining the androgen metabolome in serum, urine, and CSF in a large cohort of women with active IIH (papilledema and lumbar puncture pressure >25 cm CSF on the day of sample collection) compared with sex-, age-, and BMI-matched women with PCOS or simple obesity. We used state-of-the-art mass spectrometry–based analysis and employed cell models to investigate the functional role of androgens in CSF secretion.

## Results

### Women with IIH have a distinct androgen excess profile compared with PCOS and simple obesity.

**Figure 1:**
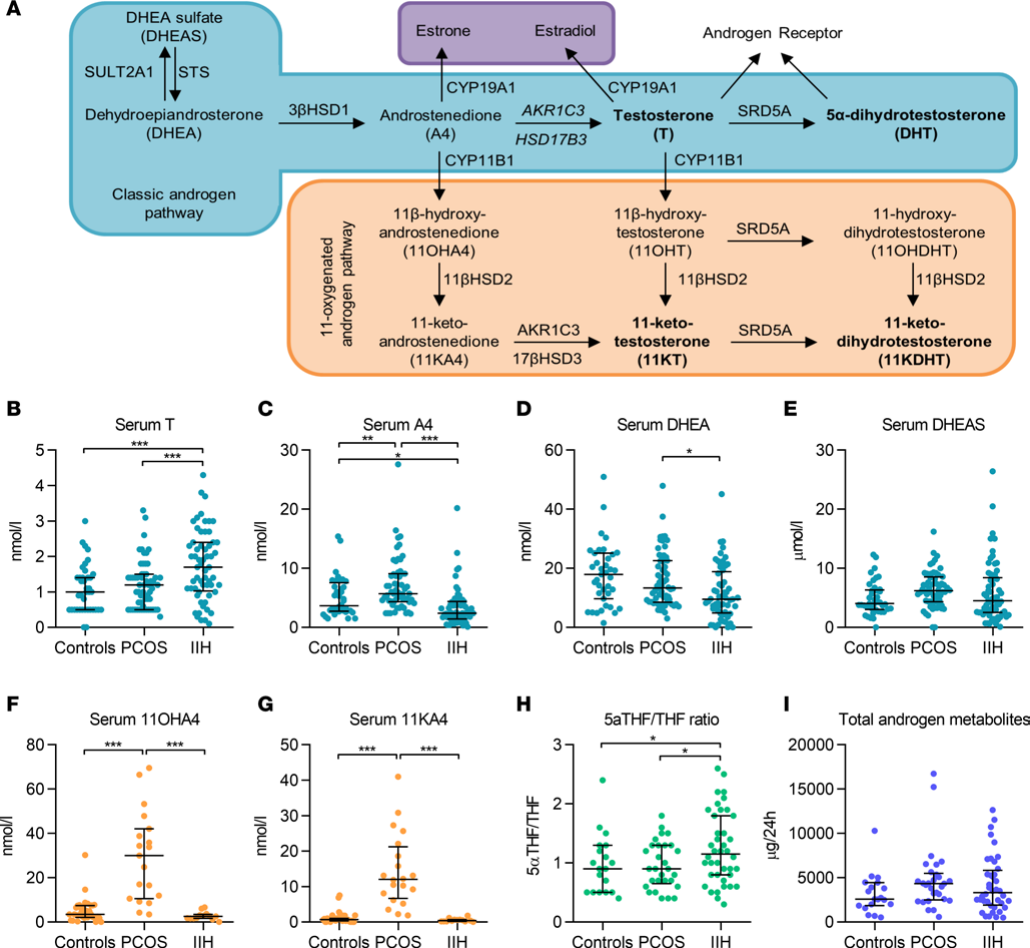
Serum and urinary androgen metabolism. (**A**) Androgen pathways. (**B**–**E**) Serum androgen concentrations in obese controls (*n* = 40), PCOS patients (*n* = 60), and IIH patients (*n* = 70). (**F** and **G**) Serum 11-oxygenated androgens in obese controls (*n* = 35), PCOS patients (*n* = 20), and IIH patients (*n* = 13). (**H** and **I**) Urinary steroid excretion in obese controls (*n* = 15), PCOS patients (*n* = 30), and IIH patients (*n* = 40). 5α-Reductase activity is indicated by the ratio of 5α-tetrahydrocortisol/tetrahydrocortisol (5α-THF/THF), and total androgen metabolite excretion was calculated as the sum of androsterone plus etiocholanolone. Data presented as median and interquartile range. *P < 0.05, **P < 0.01, ***P < 0.001; Kruskal-Wallis test.

Age and BMI were not significantly different among the 3 groups comprising women with IIH (*n* = 70), simple obesity (*n* = 40), and PCOS (*n* = 60) (Table 1). We measured serum androgens by liquid chromatography–tandem mass spectrometry (LC-MS/MS) (Table 1 and Figure 1). Serum concentrations of the active androgen testosterone (Figure 1B) were significantly higher in patients with IIH compared with controls with PCOS and simple obesity (all *P* < 0.001). Conversely, the androgen precursor androstenedione (Figure 1C) was significantly lower in IIH than in controls with PCOS (*P* < 0.001) and obesity (*P* < 0.05). In keeping with previous studies, serum androstenedione was higher in PCOS than in controls with obesity (*P* < 0.01) and IIH (*P* < 0.001). 11-Oxygenated androgens are adrenal derived and have recently been shown to be the major contributor to androgen excess in PCOS (16); therefore, we also determined their concentrations. Serum concentrations of the 11-oxygenated androgen precursors 11OH4 (Figure 1F) and 11KA4 (Figure 1G) were increased only in PCOS (all *P* < 0.001) compared with controls with IIH and obesity.

Systemic steroid metabolism in 24-hour urine samples was profiled using gas chromatography-mass spectrometry in the 3 study groups (Table 1 and Figure 1, H and I). The ratios of An/Et and 5α- 5α-THF/THF determine net systemic 5α-reductase activity, an important reaction in androgen activation (17), which was significantly increased in IIH as compared with controls with PCOS and obesity (*P* < 0.05).

### Active androgens are elevated in CSF from women with IIH compared with controls.

**Figure 2:**
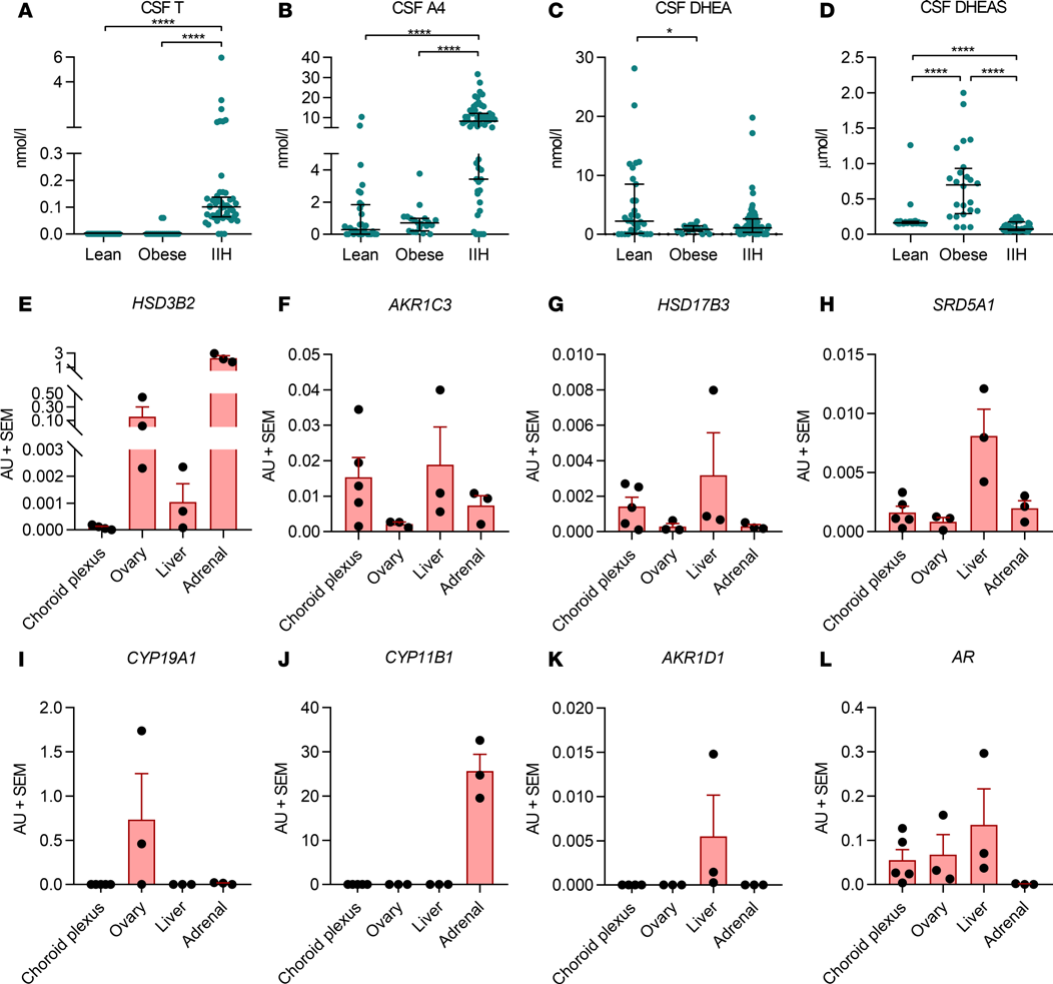
CSF androgen metabolism. Lean controls (mixed non-IIH neurological diseases) (*n* = 31), obese controls (healthy volunteers with obesity) (*n* = 19), and IIH patients (*n* = 55). (**A**) Testosterone (T) and (**B**) androstenedione (A4) were significantly higher in the IIH cohort compared with both control groups (*P* < 0.0001 for both). (**C**) CSF DHEA levels were higher in lean compared with obese controls but did not differ between controls and women with IIH. (**D**) CSF DHEAS levels were significantly higher in control women with obesity than in both lean controls and women with IIH (*P* < 0.0001). (**E**–**L**) *HSD3B2*, *AKR1C3*, *HSD17B3*, *SRD5A1*, *CYP19A1*, *CYP11B1*, *AKR1D1*, and *AR* mRNA expression in human choroid plexus (*n* = 5), ovary (*n* = 3), liver (*n* = 3), and adrenal (*n* = 3) tissue. Axes not uniform due to differences in mRNA expression. (**A**–**D**) Presented as median and interquartile range. *P < 0.05, ****P < 0.0001; Kruskal-Wallis test. (**E**–**L**) Presented as mean ± SEM.

We measured CSF androgen concentrations in 55 women with IIH, 19 healthy volunteer controls with obesity and no known comorbidities, and 31 lean controls (indications for lumbar puncture: headache *n* = 9, demyelination *n* = 6, cerebrovascular disease *n* = 3, normal investigations *n* = 5, other *n* = 8) (Figure 2, A–D). The obese controls were matched for age but had a higher BMI than the cohort with IIH (*P* = 0.01); lean controls were significantly older (*P* < 0.001) than the IIH patients (Supplemental Table 1; supplemental material available online with this article; https://doi.org/10.1172/jci.insight.125348DS1). CSF levels of the active androgen testosterone were significantly higher in IIH patients (*P* < 0.001, Figure 2A). CSF concentrations of the immediate testosterone precursor androstenedione were significantly higher in IIH patients than controls (*P* < 0.0001, Figure 2B). CSF concentrations of the androgen precursor DHEA were higher in lean compared with obese control patients (*P* = 0.04) but did not differ significantly between other groups, with CSF DHEAS highest in the obese control group and significantly greater in both lean and obese controls than in women with IIH (Figure 2, C and D, P** < 0.0001). 11-Oxygenated androgens were detectable in CSF in both IIH patients and control women but did not differ significantly between the 2 groups. Serum androstenedione and testosterone both correlated significantly with CSF androstenedione and testosterone (*r* = 0.31, *P* = 0.03; and *r* = 0.32, *P* = 0.03, respectively). Total amounts of serum and CSF androgens (calculated as the sum of testosterone, androstenedione, DHEA, and DHEAS) were also positively correlated (*r* = 0.29, *P* = 0.02).

There were no significant correlations between serum or CSF androgen concentrations and BMI or markers of IIH disease activity (ICP, papilledema, logMAR visual acuity, Humphrey Visual Field mean deviation, average and maximal retinal nerve fiber layer thickness measured by Optical Coherence Tomography [OCT], Headache Impact Test-6 [HIT-6], or subjective health status [SF-36 questionnaire]) (Supplemental Methods).

### Human choroid plexus expresses androgen receptor and androgen-activating enzymatic machinery.

IIH pathogenesis is speculated to involve either increased CSF secretion (predominantly at the choroid plexus) or reduced CSF-draining pathways (18). We have previously demonstrated that pharmacological reduction of CSF secretion can reduce ICP (19, 20). To characterize the role androgens could play in CSF secretion and potentially IIH pathogenesis, we profiled mRNA expression of androgen-metabolizing enzymes in human female choroid plexus samples (*n* = 5) (Parkinson’s UK Brain Bank, Imperial College, London, United Kingdom). These included 3β-hydroxysteroid dehydrogenase type 2 (*HSD3B2*), aldoketoreductase type 1 C3 (*AKR1C3*), 17β-hydroxysteroid dehydrogenase type 3 (*HSD17B3*), 5α-reductase type 1 (*SRD5A1*), aromatase (*CYP19A1*), 11β-hydroxylase (*CYP11B1*), aldoketoreductase type 1 D1 (*AKR1D1*), and the androgen receptor (*AR*)] (Figure 2, E–L), as well as 5α-reductase type 2 (*SRD5A2*, not shown), comparing expression levels to female human ovary, liver, and adrenal tissues (*n* = 3). Expression levels of the steroidogenic enzymes *AKR1C3* (activation of androstenedione to testosterone), *17BHSD3*, *SRD5A1*, and *AR* in the human choroid plexus samples were equivalent to or greater than expression levels observed in adrenal and ovarian tissue. Expression of *SRD5A2*, *HSD3B2*, *CYP19A1*, and *AKR1D1* was negligible or undetectable in all samples. Furthermore, *CYP11B1*, which catalyzes the conversion of the classic androgen precursor androstenedione to 11-hydroxyandrostenedione, was also not expressed in human choroid plexus (Figure 2J). This is in keeping with our finding of normal concentrations of 11-oxygenated androgens in serum and CSF of IIH patients. We have previously noted the absence of *HSD11B2* expression in human choroid plexus (7)

### Testosterone increases Na^+^/K^+^-ATPase activity in rodent choroid plexus in vitro.

**Figure 3:**
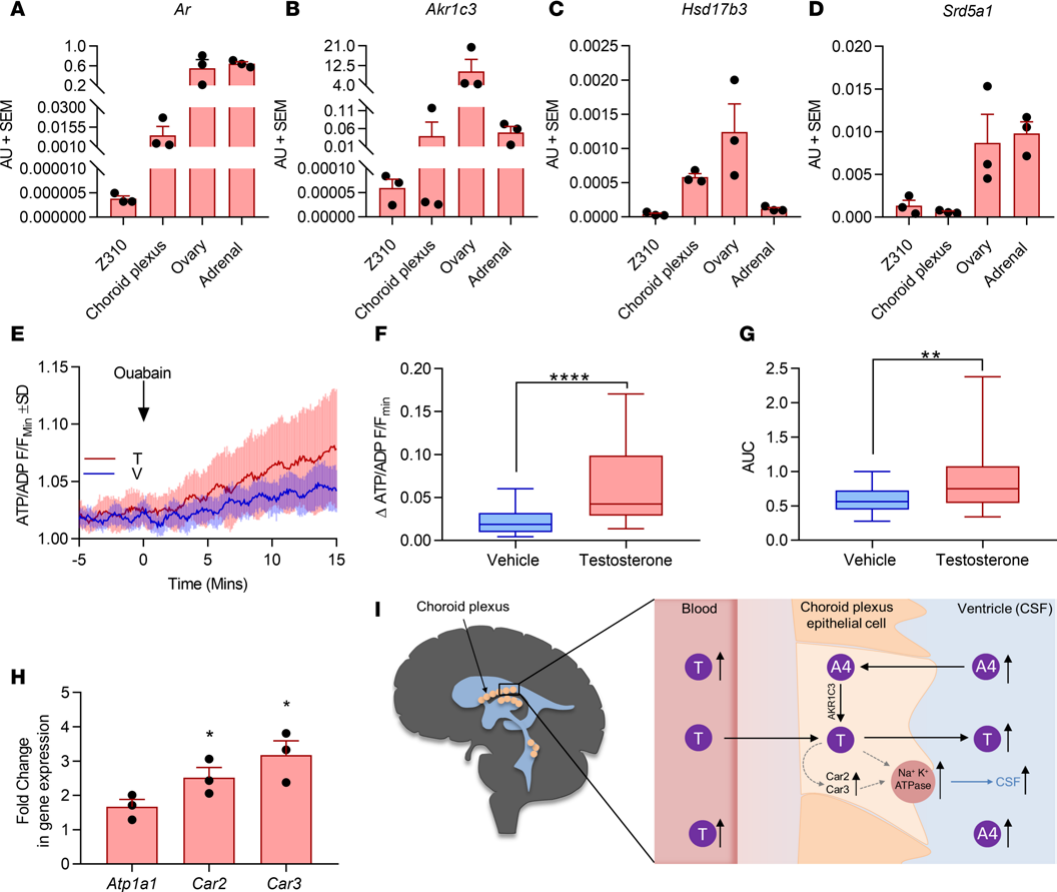
Functional effect of testosterone on rodent choroid plexus. (**A**–**D**) mRNA expression in female rats normalized to ribosomal 18S; *n* = 3 biological replicates (mean ± SEM). Z310 cells are immortalized choroid plexus epithelial cells. (**E**–**G**) Z310 cells incubated with 100 nM testosterone for 2 days increased the ΔATP/ADP ratio at 15 minutes and AUC (AU) of the ratio over 15 minutes, indicating increased Na^+^/K^+^-ATPase activity, a surrogate measure of CSF production (*n* = 39 cells from 3 pooled coverslips). V, vehicle; F, intensity for cell in particular frame; F_min_, lowest fluorescence intensity at baseline. Data presented as mean ± SD in **E**; box and whisker plots in **F** and **G** present data as median and interquartile range, showing minimum and maximum values. (**H**) mRNA expression of carbonic anhydrases II and III (*Car2* and *Car3*) and Na^+^/K^+^-ATPase (*Atp1a1*) at 48 hours in testosterone-treated (versus vehicle) Z310 cells (*n* = 3 repeats, mean ± SEM). Mann-Whitney *U* test used for **E**–**H**; *P < 0.05, **P < 0.01, and ****P < 0.0001. (**I**) Concept figure linking systemic and CSF androgen activation and impact on CSF secretion at the choroid plexus.

Akin to human choroid plexus, rat choroid plexus tissue and choroid plexus epithelial cells (Z310 cell line) expressed key steroidogenic enzymes and *Ar* (Figure 3, A–D). To determine whether testosterone could modulate CSF secretion, we used a dynamic reporter assay of ATP consumption (Supplemental Methods and Figure 1) in Z310 cells. This assay evaluates the drop in ATP/ADP ratio in the presence and absence of ouabain and hence provides a readout of the Na^+^/K^+^-ATPase activity, a validated surrogate measure of CSF secretion (20). Testosterone was found to increase the ΔATP/ADP ratio compared with vehicle-treated cells at 15 minutes (Δ0.042 ± 0.07 versus Δ0.018 ± 0.02, *P* < 0.0001) and the area under the curve of the ratio over 15 minutes (0.77 ± 0.59 versus 0.56 ± 0.28, *P* < 0.01), indicating increased Na^+^/K^+^-ATPase activity and by implication CSF production (Figure 3, E–G). Expression of carbonic anhydrases II and III (*Car2* and *Car3*), which establish the ion gradient to drive CSF secretion, were significantly upregulated (both *P* < 0.05) in testosterone-treated Z310 cells at 48 hours compared with vehicle-treated controls. Levels of Na^+^/K^+^-ATPase (*Atp1a1*) expression did not change. (Figure 3H).

## Discussion

In the current study, we provide potentially novel insights into potential mechanisms relating to the pathogenesis of IIH. We highlight a distinct profile of androgen excess in women with IIH and demonstrate an impact of androgens on surrogate markers of CSF secretion in a cell model, providing evidence for a pathogenetic link between androgen excess and increased ICP in IIH.

We show that women with IIH have a unique circulating androgen excess signature, with significantly higher active testosterone but lower concentrations of the androgen precursors DHEA and androstenedione than in women with PCOS or simple obesity. Previous studies of androgen metabolism in PCOS show complex contributions from both the classic (21) and 11-oxygenated androgen pathways (14). Women with PCOS have systemic upregulation of androgen-activating 5α-reductase activity, which converts testosterone to potent dihydrotestosterone (17), and androgen activation is enhanced in peripheral tissues, such as adipose (22). 11-Oxygenated C19 androgens are the major circulating androgens in PCOS (16). By contrast, we found that 11-oxygenated androgen concentrations in serum and CSF were not increased in IIH, indicating that adrenal androgen synthesis is not a major contributor to IIH-related androgen excess.

We measured CSF androgens using a highly sensitive and specific LC-MS/MS method. CSF testosterone concentrations were significantly higher in IIH, with relatively low levels of the inactive androgen precursor sulfate ester DHEAS. Interestingly, CSF androstenedione was significantly higher in IIH than controls, a different pattern than that observed in serum. CSF testosterone and estradiol have been previously quantified in women with PCOS using immunoassays (23); however, the absolute levels are not comparable to our data because immunoassays can be hampered by cross-reactivity, decreasing sensitivity and specificity compared with LC-MS/MS (24), particularly for quantification of steroids present in low concentrations. Different systemic and target tissue-specific hormone concentrations are a recognized phenomenon, with discrepant systemic and adipose tissue concentrations of dihydrotestosterone identified in PCOS (22). In the context of IIH, it is possible that high CSF androstenedione concentrations provide a pool of androgen precursors for activation to testosterone by the choroid plexus (Figure 3I). In this study, we found expression of the androgen-activating enzyme *AKR1C3* in human choroid plexus, indicating the presence of the enzymatic machinery required for activation of androstenedione to testosterone. Significant aromatization of androstenedione to estrone, or testosterone to estradiol, within the choroid plexus appears unlikely in view of the low levels of choroid plexus *CYP19A1* expression and the well-recognized poor efficiency of this enzyme in converting androgens to estrogens (25).

CSF is in intimate contact with the choroid plexus; therefore, CSF androgen excess could affect choroid plexus function. We have shown that human choroid plexus expresses both the AR and the critical steroidogenic machinery for androgen activation, but whether androgens are activated directly in the choroid plexus, or reach the choroid plexus after activation in the peripheral tissues, such as adipose, remains unclear. Using Na^+^/K^+^-ATPase activity as a surrogate of CSF secretion, we demonstrated that testosterone could drive CSF output in choroid plexus cells. These observations are supported by increased mRNA expression of *Car 2* and *Car 3* after testosterone exposure, providing evidence for a role of androgens in modulating CSF dynamics in IIH.

A central role for androgens in the pathogenesis of IIH in women may seem biologically implausible when it is considered that IIH is rare in men (5). However, androgens exert sexually dimorphic effects on human metabolism (26). Men with hypogonadism develop a similar adverse metabolic phenotype to women with androgen excess. In both groups, the risk of type 2 diabetes, nonalcoholic fatty liver disease, and cardiovascular mortality are increased (27, 28). There are a number of potential explanations for this sex-specific difference, including epigenetic modifications to local androgen action or differences in the sensitivity or signaling of the AR in both sexes. IIH may therefore be a distinct manifestation of these sexually dimorphic observations. Intriguingly, men with IIH are more likely to have symptoms of androgen deficiency, such as erectile dysfunction and reduced libido (29). Additionally, men can develop an IIH-like condition (Pseudotumor cerebri) in the setting of low testosterone attributed to gonadotropin deficiency or administration of androgen deprivation therapy (30).

Our study is limited in that it has not characterized androgen metabolism in a male cohort with IIH. This would be of interest but these studies are challenging because male IIH is very rare, accounting for less than 5% of all IIH cases (5). Additionally, IIH in men is atypical, often caused by secondary factors, and may not share a similar etiology to IIH in women (3).

The relationship between testosterone and body weight is of interest. Our studies are limited because we cannot exclude the possibility that androgen excess has driven weight gain in the obese participants. Androgen excess can increase visceral adiposity; however, it can also increase lean mass, though these effects are typically modest. BMI only increased from 29.1 to 30.0 kg/m^2^ in a study of a female-to-male sex reassignment patients 6 months after initiation of testosterone (31). To minimize the impact of weight influencing our findings, we compared the androgen profiles in serum, urine, and CSF to controls matched for BMI. Additionally, we noted the absence of a correlation between these measures and BMI in the IIH cohort. Future evaluation of the effects of weight loss on androgen profiles and disease activity would be useful to clarify this relationship further.

Using a cell-based model, we have demonstrated the potential for androgens to modify CSF secretion in this study. The relative importance of CSF oversecretion versus CSF underdrainage is debated in the literature (18). It is unlikely that dysregulation of either CSF secretion or drainage occurs in isolation, and indeed these processes are likely to influence each other. Our study is limited in that we have not assessed the impact of androgens on CSF drainage.

Current therapeutic strategies for IIH are limited (5), with significant morbidity from visual loss and long-term headache. We show that women with IIH have a distinct androgen excess signature, which, with the in vitro assay on Na^+^/K^+^-ATPase activity, supports a potential role for androgens in the pathogenesis of IIH. We propose that potent androgens are activated in the choroid plexus and/or cross the blood-brain barrier after activation in other tissues where they affect CSF secretion and ICP. Therapeutically targeting androgen excess in IIH will be an important next step in testing this hypothesis in the clinical setting.

## Methods

Detailed descriptions of experimental procedures are reported in the Supplemental Methods.

### Cohorts.

Women with IIH aged between 18 and 45 years were recruited from neurology clinics at University Hospital Birmingham, as well as ophthalmology clinics at the Birmingham and Midland Eye Centre, after they provided written informed consent. IIH patients were diagnosed according to the modified Dandy criteria and were included only if they had active, untreated disease with ongoing papilledema (Frisen grade ≥1; ref. 32) and opening CSF pressure greater than 25 cm H_2_O at the time of sample collection (1).

Women with PCOS as well as healthy controls were recruited from endocrine outpatient clinics at University Hospital Birmingham and Birmingham Women’s Hospital and from local advertising, respectively, with full ethical approval obtained (see Study Approval in main Methods section). A further cohort of healthy control women was recruited from Adelaide and Meath Hospital, Dublin, Ireland. PCOS was diagnosed according to the Rotterdam European Society of Human Reproduction and Embryology 2004 criteria (presence of 2 or more of the following criteria: oligo-anovulation, clinical or biochemical hyperandrogenism, and polycystic ovaries on ultrasound).

The female lean control cohort for CSF evaluation consisted of patients with non-IIH neurological disease (after providing written informed consent) and a control cohort with obesity (with no known comorbidities) recruited solely for research evaluation through local advertising. Written informed consent was provided in all cases.

Exclusion criteria for the study were recent glucocorticoid therapy (within 3 months), pregnancy, recent oral contraceptive or antiandrogen use (within 3 months), hyperprolactinemia, and overt hyperglycemia. PCOS was excluded on clinical and biochemical assessment from the control cohorts. The cohorts were whole-group matched (for age, sex, and BMI) for comparison unless otherwise stated. Participants attended the NIHR/Wellcome Trust Clinical Research Facility at University Hospital Birmingham after an overnight fast. Baseline anthropometric data were collected and serum samples were drawn for measurement of serum androgens. Precollected 24-hour urine samples for urinary steroid metabolite profiling were also provided by patients upon arrival to the research facility. In IIH patients, CSF samples were also collected by lumbar puncture (LP). CSF and blood samples were transported on ice before being promptly centrifuged at 226 x *g* for 10 minutes and aliquoted. All samples were stored at –80°C and analyzed after a maximum of 1 freeze-thaw cycle.

Visual assessments were recorded during the research visit. Best corrected visual acuity was measured with a Humphrey Visual Field analyzer using the Swedish Interactive Threshold Algorithm Standard 24-2 program and mean deviation was recorded. OCT (Heidelberg Spectralis Spectral Domain OCT) was acquired to record average and maximal retinal nerve fiber layer thickness. LP was performed after all visual assessments. LP pressure was evaluated with the participant breathing steadily in the lateral position, legs flexed 90° at the hip, with adequate time taken to ensure a stable reading. Headache disability was recorded using the HIT-6 (33). Quality of life was assessed using the SF-36 Version 1 (RAND 36-Item Short Form Survey).

### ATP/ADP CSF secretion assay.

CSF secretion occurs predominantly at the choroid plexus epithelial cells; numerous ion channels are involved but the Na^+^/K^+^-ATPase is a key rate-limiting step in driving CSF secretion (34, 35). Specific inhibition of the Na^+^/K^+^-ATPase with ouabain reduces CSF secretion by 70% to 80% (35). ATP consumed by Na^+^/K^+^-ATPase activity is a marker of CSF secretion (20). Na^+^/K^+^-ATPase activity was measured by determining increases in the ATP/ADP ratio in the presence and absence of ouabain (35). Measures of the total ATP/ADP could not be used because they reflect multiple cellular processes and are not specific for Na^+^/K^+^-ATPase activity; hence we report only the changes in the ATP/ADP ratio sensitive to ouabain.

Assays were conducted in Z310 cells (courtesy of Wei Zheng, Purdue University, West Lafayette, Indiana, USA), an immortalized cell line derived from primary rat choroid plexus epithelium transfected by simian virus 40 T antigen (36). Cells were infected 2 days before imaging with adenovirus containing Perceval, an ATP/ADP ratio biosensor. The ATP/ADP ratio was imaged using a CrestOptics X-Light spinning disk head coupled to a Nikon Ti-E automated base and 10×/0.4× numerical aperture objective. Excitation was delivered by a Lumencor Spectra X light engine at 458–482 nm (0.2 Hz), and emitted signals were detected with a Photometrics Evolve Delta 512 EMCCD at 500–550 nm. The ATP/ADP traces were normalized as F/F_min_, where F is the fluorescence at a given time point and F_min_ is the minimum fluorescence. Cells were treated with either testosterone (100 nM) or acetazolamide (100 mM; positive control) before recording ATP/ADP and applying 1 mM ouabain, the increase in Perceval fluorescence reflecting the portion of ATP consumed by Na^+^/K^+^-ATPase activity. A HEPES-bicarbonate buffer was used, containing the following in mM: 120 NaCl, 4.8 KCl, 24 NaHCO_3_, 0.5 Na_2_HPO_4_, 5 HEPES, 2.5 CaCl_2_, 1.2 MgCl_2_, and 25 D-glucose.

### Analysis of live cell data.

Images were acquired with MetaMorph (Molecular Devices). An image sequence was initially analyzed on ImageJ (V1.48; NIH), where individual random cells are selected as a region of interest and motile cells are excluded from the analysis. Following this, the multimeasure function was selected to determine the mean intensity of individual cells at each frame. Subsequent to this the F/F_min_ was determined for each frame, where F is the intensity for the cell in that particular frame and F_min_ is the lowest fluorescence intensity in the baseline period. This normalizes the change intensity to baseline, taking into account any quantitative difference in ATP/ADP ratio between cells or differences in Perceval protein expression. We used ouabain administration to initiate a change in ATP/ADP ratio or Δ (Supplemental Figure 1). This Δ is the maximum F/F_min_ value following ouabain administration minus the maximum F/F_min_ in the baseline (before ouabain administration).

### Statistics.

Statistical analysis was performed using SPSS Statistics (Version 22, IBM, Chicago, Illinois, USA). All comparisons were performed using Student’s *t* test (2-tailed), Mann-Whitney *U* test, or Kruskal-Wallis testing. *P* values < 0.05 were deemed significant.

### Study approval.

For procedures involving PCOS and healthy controls, approval was granted by the South Birmingham (LREC5835), Edgbaston (12/WM/0206), and Adelaide and Meath Hospital Dublin (2006/10/02) Research Ethics Committees. Ethical approval for recruitment of IIH patients was granted by the Dudley (06/Q2702/64), Yorkshire and the Humber–Leeds West (13/YH/0366), and Black Country (14/WM/0011) local Research Ethics Committees**.** Ethical approval for CSF samples from patients with neurological disease was granted by Solihull Research Ethics Committee (04/Q2706/65). The Parkinson’s UK Brain Bank has ethical approval from the Wales Research Ethics Committee as a research tissue bank, which covers the use of tissue from the Brain Bank by researchers.

## Author contributions

AJS, MWO, and WA conceptualized and designed the clinical study. All authors were involved in aspects of study conduct as well as drafting and approving the final manuscript.

## Supplementary Material

Supplemental data

## Figures and Tables

**Table 1:**
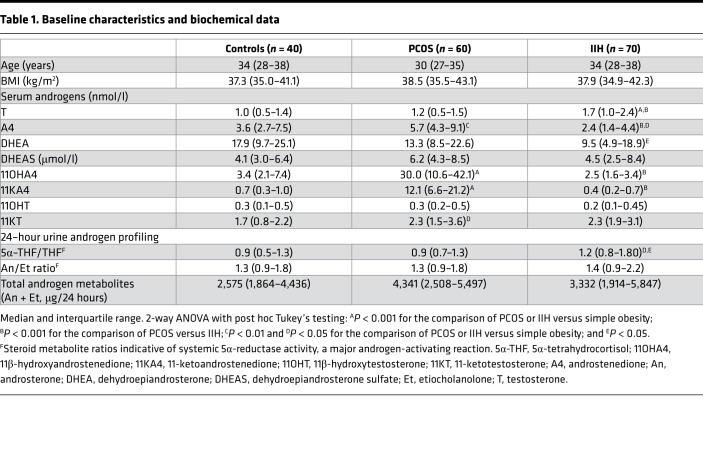
Baseline characteristics and biochemical data

## Version 1 02/12/2019

In-Press Preview

## Version 2 03/21/2019

Electronic publication

