# Supplementary material for: A unique androgen excess signature in idiopathic intracranial hypertension is linked to cerebrospinal fluid dynamics
Source: JCI Insight. 2019 Mar 21;4(6):e125348. doi: 10.1172/jci.insight.125348 (PMC6483000; doi:10.1172/jci.insight.125348)
Supplement: Supplemental data [file jciinsight-4-125348-s015.pdf]

SUPPLEMENTAL DATA

RESULTS

***Supplemental Table 1*:** Baseline characteristics of CSF study patients. Data are presented as median and interquartile range. Statistical comparison was carried out by analysis of variance with *post hoc* Tukey testing. Significance levels were indicated as follows: a, p<0.05, b, p<0.01, c, p<0.001 for the comparison of controls v IIH.

	**Lean controls (n=31)**	**Obese controls (n=19)**	**IIH (n=55)**
Age (years)	46 (38-57)^c	37 (32-46)	30 (27-35)
BMI (kg/m^2)	27 (22.7-32.3)^c	44.8 (39.7-47.3)^c	37.9 (34.9-42.3)
LP pressure (cm H_20)	16.5 (13.5-20.8)^c	23.5 (21-25.5)^c	36.0 (29.5-40.0)

Abbreviations: BMI, body mass index; IIH, idiopathic intracranial hypertension; LP, lumbar puncture

METHODS

Steroid analysis

All serum and CSF steroids were measured by tandem mass spectrometry (LC-MS/MS) as previously described (1). DHEAS was extracted from 20 µL of serum and CSF by the addition of 20 µL 0.1 mM $ZnSO_4$ and 100 µL acetonitrile and subsequently quantified in negative mode using a Xevo TQ mass spectrometer (Waters, Milford, USA) coupled to an ACQUITY UPLC system (Waters, Milford, USA) (4). All other steroids were extracted from serum and CSF (400 µL for each) using 2 mL Methyl *tert*-butyl ether (MTBE) as previously described. Serum T, A4, DHEA, 11OH4, 11OHT and 11KT, as well as CSF T, A4 and DHEA, were quantified using a Xevo TQ-S mass spectrometer (Waters, Milford, USA) coupled to an ACQUITY UPLC system (Waters, Milford, USA). The serum steroids were separated using a UPLC high strength silica (HSS) T3 column (2.1 mm x 50 mm, 1.8 µm) (Waters, Milford, USA) using water 0.1% formic acid (A) and methanol 0.1% formic acid (B) as mobile phases. Separation was achieved using a 5 min linear gradient from 55% A to 75% B at a constant flow rate of 0.6 mL min^{-1} and a column temperature of 50°C. All steroids were analysed in multiple reaction monitoring (MRM) using the settings reported by Quanson *et al*. Serum 11KA4 was quantified using

an ACQUITY UPC^2 system (Waters, Milford, USA) coupled to a Xevo TQ-S mass spectrometer (Waters, Milford, USA) as previously reported. Data collection and analysis were performed using MassLynx 4.1 (Waters, Milford, USA). Validation data has previously been published by our group (5).

Urinary steroid metabolite profiling was performed by gas chromatography-mass spectrometry (GC-MS) in selected ion-monitoring (SIM) mode as previously described (2). The ratios of 5alpha-reduced steroid over 5beta-reduced steroids is an established measure of systemic 5a-reductase activity (3), which we determined by calculating the ratios of the androgen metabolites androsterone (An) over etiocholanolone (Et) and the glucocorticoid metabolites 5α-tetrahydrocortisol (5α-THF) over tetrahydrocortisol (THF). Total urinary androgen metabolite excretion was calculated by the sum of An and Et.

mRNA expression analysis

Human choroid plexus tissue was obtained from the Parkinson's Brain bank at Imperial College London, UK. Total RNA was extracted from human choroid plexus tissue and Z310 cells using the commercially available GenElute Mammalian Total RNA Miniprep Kit (Sigma-Aldrich Ltd, Dorset, UK). Complementary DNA (cDNA) was produced by reverse transcription of RNA using the Applied Biosystems High Capacity cDNA Reverse Transcription Kit according to manufactures instructions.

qPCR was performed using the Applied Biosystems 7500 system. Taqman Gene Expression Assays (Life Technologies) were used to assess expression of a number of androgen metabolism enzymes (see supplemental material for full list of genes and assay numbers). Reactions were carried out in 96 well plates, singleplex 10μl reaction volumes, using Taqman Gene Expression Master Mix (Applied Biosystems). Human choroid plexus and rat tissue reactions were internally controlled using the endogenous *18S* ribosomal subunit as the reference (Applied Biosystems, 4319413E). Primers are provided in the **Supplemental Table 2**. Data was expressed in arbitrary units (AU), calculated using the transformation $1000*2^{-\Delta Ct}$, where ΔCycle Threshold (ΔCt) = [Ct of target gene] – [Ct of internal reference gene (internal reference)].

Supplemental Table 2

**Gene**	**Species**	**Code**	**Dye**
18S	Eukaryotic	4319413E	VIC
AR	Human	Hs00171172_m1	FAM
AKR1C3	Human	Hs00366267_m1	FAM
CYP19A1	Human	Hs00903411_m1	FAM
HSD3B2	Human	Hs00605123_m1	FAM
SDR5A1	Human	Hs00602694_m1	FAM
HSD17B3	Human	Hs00970004_m1	FAM
Ar	Rat	Rn00560747_m1	FAM
Akr1c3	Rat	Rn00684527_m1	FAM
Car2	Rat	Rn01462065_M1	FAM
Car3	Rat	Rn01461970_M1	FAM
			FAM
			FAM
			FAM
Hsd17b3	Rat	Rn00588942_m1	FAM
Srd5a1	Rat	Rn00567064_m1	FAM
Atp1a1	Rat	Rn01533986_m1	FAM

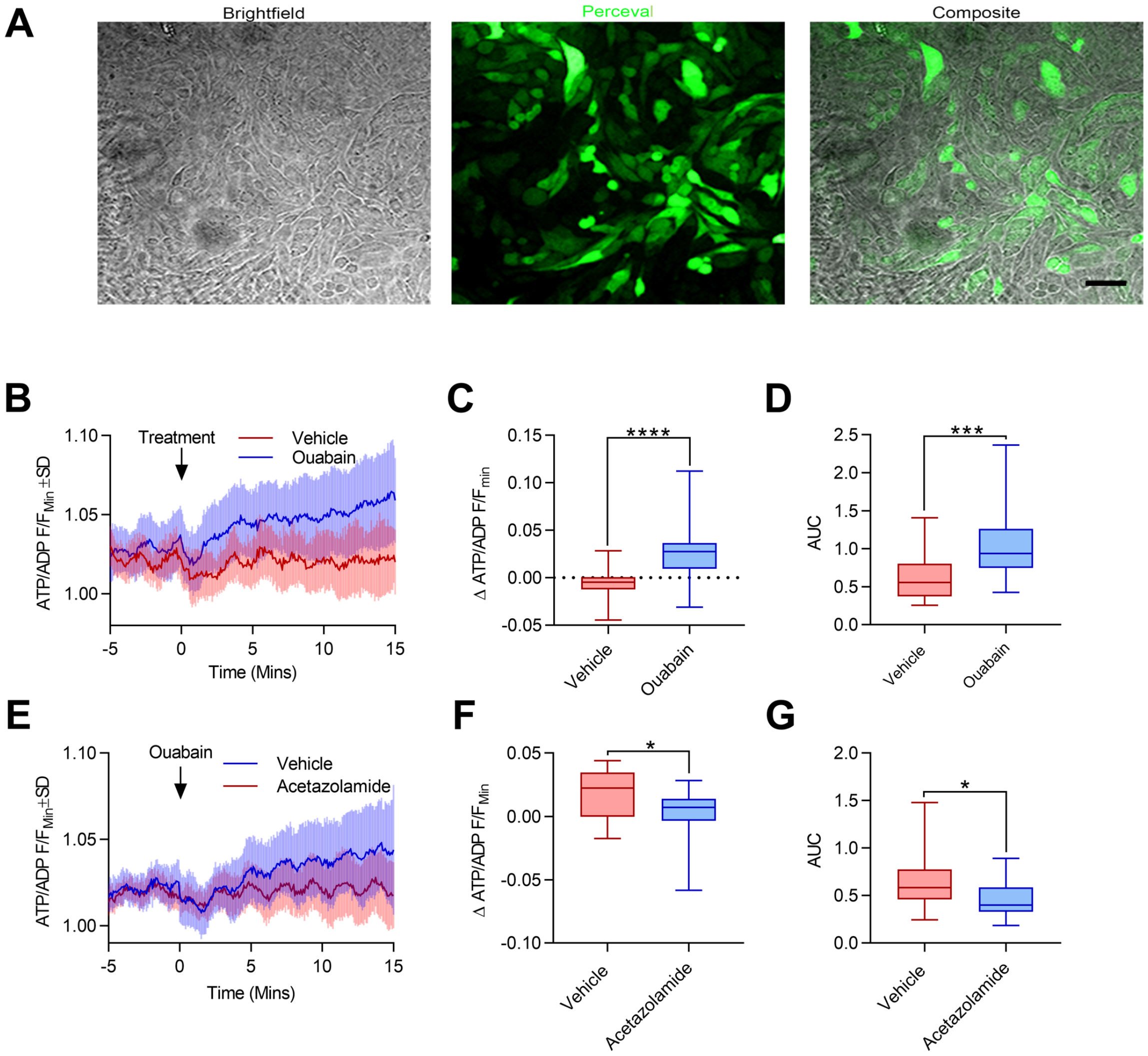

Supplemental Figure 1: Development and optimisation of a novel Na/K ATPase assay

(A) Confluent Z310 cells express Perceval two days post adenovirus infection. (B) Trace detailing the normalised ATP/ADP ratio overtime in vehicle treated (red) and ouabain treated (blue) cells. (C) Ouabain changes the maximum F/Fmin significantly [Δ0.027 (0.026)], compared to vehicle [Δ-0.005 (0.012), p<0.0001)], indicating that the assay can measure Na/K ATPase activity (D) The area under curve (AUC) derived from both lines on B, where ouabain is higher [0.93 (0.5171)] compared to vehicle [0.55 (0.4336), p<0.001]. (E) Perceval F/Fmin trace of vehicle treated cells or cells treated with acetazolamide (100mM). (F) The change in ATP/ADP maximum following ouabain in acetazolamide-treated cells [0.007 (0.01039)] is reduced compared to untreated cells [0.2404 (0.031778), p=0.029], detailing that the assay can measure manipulated Na/K ATPase activity. (G) The area under the curve

of acetazolamide treated cells is lower [0.4473 (0.3452)] compared to no treatment [0.61 (0.38), p=0.044)]. (E) Trace is presented as mean ± SD; C, D, F, G, data presented as median (IQR, minimum and maximum values), for both vehicle and ouabain treated cells N=27 cells from 3 pooled coverslips. Scale bar =50 microns. Mann-Whitney test for B-G, *P<0.05, ***P<0.001 and ****P<0.0001.

Supplemental references

1. O'Reilly MW, Taylor AE, Crabtree NJ, Hughes BA, Capper F, Crowley RK, Stewart PM, Tomlinson JW, and Arlt W. Hyperandrogenemia predicts metabolic phenotype in polycystic ovary syndrome: the utility of serum androstenedione. *The Journal of clinical endocrinology and metabolism.* 2014;99(3):1027-36.
2. Shackleton C, Marcos J, Arlt W, and Hauffa BP. Prenatal diagnosis of P450 oxidoreductase deficiency (ORD): a disorder causing low pregnancy estriol, maternal and fetal virilization, and the Antley-Bixler syndrome phenotype. *Am J Med Genet A.* 2004;129A(2):105-12.
3. Stewart PM, Shackleton CH, Beastall GH, and Edwards CR. 5 alpha-reductase activity in polycystic ovary syndrome. *Lancet.* 1990;335(8687):431-3.
